# Development of a standard set of key work-related outcomes for use in practice for patients with cardiovascular disease: a modified Delphi study

**DOI:** 10.1186/s41687-024-00825-6

**Published:** 2024-12-18

**Authors:** Marije E. Hagendijk, Nina Zipfel, Jan L. Hoving, Marijke Melles, Philip J. van der Wees, Sylvia J. van der Burg-Vermeulen

**Affiliations:** 1 Department of Public and Occupational Health, Coronel Institute of Occupational Health, Amsterdam Public Health Research Institute, Amsterdam UMC Location University of Amsterdam, Meibergdreef 9, Amsterdam, 1105 AZ The Netherlands; 2 Research Centre for Insurance Medicine, Amsterdam, The Netherlands; 3 Faculty of Industrial Design Engineering, Delft University of Technology, Delft, The Netherlands; 4 Scientific Institute for Quality of Healthcare (IQ Healthcare), Radboud University Medical Centre, Nijmegen, The Netherlands

**Keywords:** Patient-reported outcome measures, Value-based healthcare, Cardiovascular diseases, Occupational health, Work engagement, Patient-centred care, Patient preference, Interdisciplinary communication, Return to work, Workload

## Abstract

**Background:**

To facilitate the maintenance or resumption of participation in work for patients with cardiovascular disease (CVD), there is a need for high-quality work-focused healthcare. According to the concept of value-based healthcare, quality of care can be enhanced by understanding the outcomes that matter most to patients. However, a major challenge in assessing quality of work-focused healthcare in practice is the lack of consensus on which work-related outcomes should be measured.

**Objective:**

The objective of this study was to identify a standard set of key work-related outcomes for patients with CVD to be used in practice of work-focused healthcare in the Netherlands, including standardised outcome measures and associated case mix factors. This standard set is intended to assist occupational and other health professionals in delivering work-focused healthcare that meets a patient’s individual needs regarding work participation, and to enhance patients’ engagement in their own work-focused care process.

**Methods:**

A 2-round RAND-modified Delphi process was conducted. The process included literature searches, consecutive research team meetings, and several meetings and rounds of voting by a working group. The working group consisted of patients with CVD (*n* = 6) and health professionals representing different stakeholders (*n* = 11) involved in work-focused healthcare for this patient population in the Netherlands. Consensus was reached over four phases: (1) establishing the scope of the standard set and defining the population, (2) prioritising and defining the outcome domains, (3) selecting the outcome measures for the most important domains, including clinical data and patient-reported data, and (4) selecting and defining case mix factors.

**Results:**

A 23-item patient-reported questionnaire was developed, called the Value@WORK-Q23, including questions on nine work-related outcome domains considered most important for patients with CVD: (1) work participation, (2) physical work ability, (3) mental work ability, (4) suitable work, (5) support from the work environment, (6) flexibility of the work environment, (7) communication with the patient, (8) person-centredness, and (9) interdisciplinary communication. In addition, nine case mix variables was selected, comprising demographic-, disease-, and work factors.

**Conclusions:**

The Value@WORK-Q23 provides guidance on measuring the most important work-related outcomes for patients with CVD. Using this work-related set in practice, in addition to existing disease-specific standard sets for CVD may facilitate the provision of high-value work-focused healthcare for this patient population.

**Supplementary Information:**

The online version contains supplementary material available at 10.1186/s41687-024-00825-6.

## Plain English version of the abstract

**Why?:**

People with heart disease often need help to stay at work or return to work. To provide the best care, it’s important to know what work-related outcomes matter most to these patients. However, there’s no agreement on which outcomes to measure.

**What was the goal?:**

This study aimed to create a standard set of important work-related outcomes for heart disease patients in the Netherlands. This set will help health professionals provide better, personalized care and involve patients more in their own care.

**How?:**

The study used a structured process involving literature reviews, team meetings, and multiple rounds of discussions and voting with a group of patients and health professionals. The group included 6 patients with heart disease and 11 health professionals from various fields.

**What did we find?:**

The study developed a 23-question survey (the Value@WORK-Q23) for patients, covering nine key areas: work participation, physical and mental work ability, suitable work, support and flexibility at work, communication with the patient, person-centred care, and teamwork among health professionals. It also identified nine factors that could affect these outcomes, such as demographics, disease specifics, and work conditions.

**Conclusion?:**

This new survey helps measuring the most important work-related outcomes for heart disease patients. Using this survey alongside existing heart disease care standards can improve the quality of work-focused healthcare for these patients.

## Background

With the rise in the legal retirement age across most industrialised countries, the prevalence of cardiovascular disease (CVD) among the working age population is steadily increasing [1, 2]. When working age individuals are diagnosed with CVD, one of their primary concerns is whether they can continue working [3, 4]. Consequently, a decrease in the ability to work negatively affects the overall perception of their quality of life [5]. To prevent this, return-to-work or stay-at-work has been recognized as crucial indicators for general health, mental health and physical, social and emotional functioning [5]. Healthcare services that target work participation play a vital role in supporting patients with CVD in achieving a return to work or staying at work [6]. Many healthcare professionals can be involved in providing work-focused healthcare services, assessing a patient’s abilities and limitations related to work participation, and providing advice and support for functional recovery [7]. However, despite the importance of work-focused healthcare in practice, its impact remains uncertain as professionals lack knowledge on how to deliver effective work-focused healthcare [8–10]. Therefore, the needs of working-age patients with CVD are not being consistently met [7, 11].

According to the concept of value-based healthcare, quality of care can be improved by focusing on those outcomes that matter most to patients [12]. In value-based healthcare, outcomes are defined as the results of care in terms of the patient’s health over time, in contrast to care processes or interventions designed to achieve the results [13]. Measuring person-centred outcomes, including key outcomes related to the patient’s context and surroundings, can improve quality of care at both aggregate and patient level [14]. Measuring outcomes at an aggregate level is used for benchmarking, enabling learning and improving across healthcare institutions [15]. At an individual level, person-centred outcomes reported by the patient are used as input during healthcare consultations, to support shared decision-making and to discuss the patient’s needs [16–18]. A key challenge in improving the quality of work-focused healthcare in practice is the absence of consensus on which person-centred outcomes should be measured and how this should be done [19]. Therefore, there is a need for standardisation of person-centred work-related outcomes to enhance the delivery of high-value work-focused healthcare for all working-age patients with CVD.

Current research has focused on the development of an international generic core outcome set for work participation, seeking consensus on outcomes measuring the effects of interventions on work participation in intervention trials using the Core Outcome Measures in Effectiveness Trials methodology [20]. However, this generic core outcome set was developed primarily for research purposes to evaluate the effectiveness of interventons on outcomes such as return to work and work status, designed to be applied to all health conditions. Additionally, this generic core outcome set was not developed for use in work-focused healthcare practice, and does not address the broad range of needs of patients in work-focused healthcare. The International Consortium for Health Outcomes Measurements (ICHOM) has developed standard sets of person-centred outcomes, targeting key outcomes for various medical conditions, including coronary artery disease [21]. However, we found that these ICHOM sets primarily focus on disease-specific key outcomes, in which work is often either not included at all or only addressed through a single outcome domain on work functioning.

The objective of this study was to develop a standard set of key work-related outcomes for patients with CVD. This set includes standardised outcome measures and a minimal set of associated case mix factors. The goal is to facilitate work-focused healthcare practices while minimising the registration burden by targeting a minimal set [14]. This standard set of work-related outcomes can complement existing disease-specific standard sets.

## Methods

### Design and setting

**Fig. 1:**
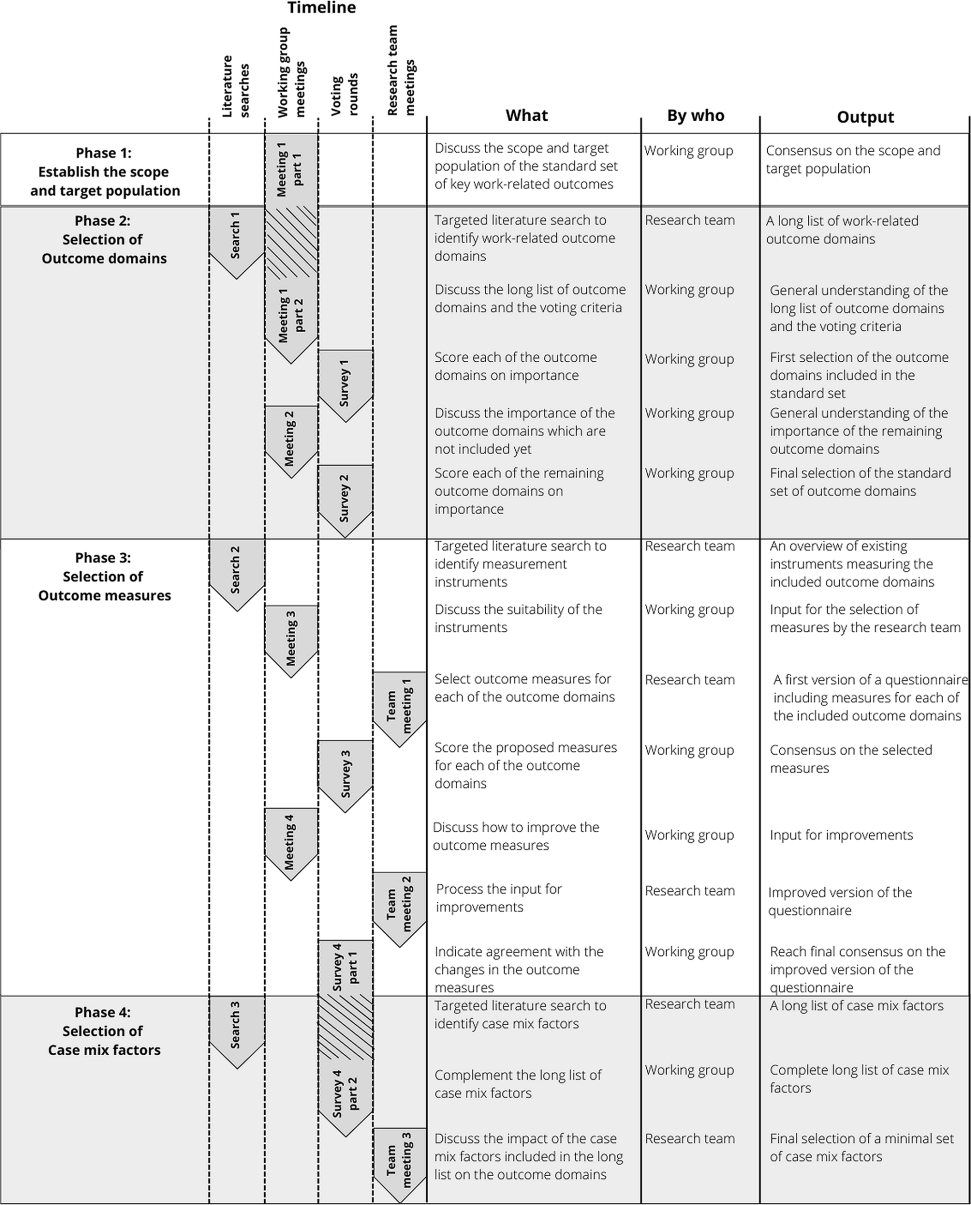
Overview of steps taken over the four phases of data collection and analysis

For the development of this standard set, the approach used by ICHOM to developing person-centred standard sets was followed [21, 22]. A 2-round modified Delphi process was conducted, following the RAND/University of California at Los Angeles methodology [23]. Consensus was reached over four phases (see Fig. 1) including working group debate: (1) establishing the scope of the standard set and defining the population, (2) prioritising and defining the outcome domains, (3) selecting the outcome measures for the most important domains, including clinical data and patient-reported data, and (4) selecting and defining case mix factors [24]. This study is conducted in the context of the Dutch healthcare system. More information on the work-focused healthcare system in the Netherlands can be found in this study [7].

### Working group composition and recruitment

Our aim was to establish an interdisciplinary working group encompassing a broad spectrum of specialities in work-focused healthcare, as outlined in the ICHOM approach [21, 22]. The people invited to join the working group were representatives of healthcare professionals involved in work-focused healthcare and patients [7]. These specialities included: insurance physicians, occupational physicians, physiotherapists, labour experts, psychologist, cardiologists, general practitioners, and patients with CVD. Prospective members were informed and invited to participate through various channels, including personal invitations via the network of the research team, open calls on social media, and invitations extended to members of associations representing the interests of the different stakeholder groups, such as the Dutch Association for Insurance Medicine, Dutch Association for Heart, Vascular and Pulmonary Physiotherapy, and the Dutch Patient Federation. Invitees who expressed interest in participating were contacted by the first author (MH) by phone to discuss the aims of the research and the obligations associated with participation.

### Data collection and analysis

To facilitate the process of debate and consensus during the four phases shown in Fig. 1, three literature searches and three meetings were undertaken by the research team, and a combination of four meetings and four voting rounds by the working group were conducted. The four working group meetings were held between February and September 2023, comprising one two-hour face-to-face meeting and three one-and-a-half hour online meetings. All meetings were chaired by either the first or second author (*n* = 3 by MH, *n* = 1 by NZ) and were supported by at least two team members in varying compositions (NZ, JH, PW, SB). Each working group meeting was followed by an online vote, administered through questionnaires created on the Microsoft Forms platform. Each meeting and voting round was supported by a poster or booklet presenting the results of the literature searches, minutes of previous meetings, proposed discussion points and/or results of the preceding voting round. The final standard set, including all measures as well as the case mix factors, was shared with the working group for their final approval. Afterwards, the final version of the standard set -intended to be completed by the patient- was checked by a professional writer to ensure B1 language level.

#### Phase 1. Establishing the scope and target population

The proposed scope of the standard set was to identify a standard set of work-related outcomes most important for patients, with the dual objective of (1) assisting healthcare professionals in meeting individual patient needs related to work participation and (2) enhancing patient engagement in their own work-focused care process. The proposed target population comprises patients of working age living with CVD. During the first meeting the working group deliberated upon the proposed scope and the target population.

#### Phase 2. Selection of outcome domains

A targeted literature search was conducted by the research team in order to provide a long list of outcome domains extracted from literature and guidelines (see Fig. 1, search 1 and supplementary material 1). To present the long list to the working group in a more structured way, the domains were organised into categories based on a previous subdivision of the workload and reintegration possibilities factor from the ICF model [25]. After discussing the long list of outcome domains and voting criteria established by the research team (see Fig. 1, meeting 1), the working group was tasked with rating each of the outcome domains on a 9-point Likert scale ranging from 1 -not important at all- to 9 -very important- (see Fig. 1, survey 1). The four voting criteria were as follows: (1) The outcome domain has a significant impact on the work participation of patients with CVD and/or on the patient’s awareness and engagement with their work-orientated care process, (2) the outcome domain can be influenced by healthcare professionals involved in work-focused healthcare, (3) the outcome domain has the potential to be measured, and (4) the outcome domain influences societal costs. Outcome domains that were rated as ‘very important’ (7–9 points) by more than 70% of the working group were promptly included in the standard set. Outcome domains falling within the 30–70% range during the first voting round were discussed at the second working group meeting. Outcome domains rated as ‘very important’ by less than 30% of working group were immediately excluded. Likewise, during the second voting round, all outcome domains rated as ‘very important’ by more than 70% of the working group were included in the standard set, while outcome domains rated as ‘very important’ by less than 70% of the working group were excluded.

#### Phase 3. Selection of outcome measures

To provide an overview of existing measurement instruments for each of the included outcome domains, a targeted literature search was conducted by the research team (see Fig. 1, search 2 and supplementary material 1). Upon reviewing the overview of existing measurement instruments, the working group discussed the suitability of these instruments for each of the outcome domains (see Fig. 1, meeting 3). During these discussions, greater emphasis was placed on selecting standardised instruments and efforts was made to retain as many original question and response options as possible. Taking into account this discussion, the research team formulated a proposal on how to measure each of the outcome domains (see Fig. 1, research team meeting 1). Then, at the third voting round, the members of the working group were asked to rate the proposed outcome measures on the 9-point Likert scale, considering four voting criteria: (1) the suitability of the outcome measure for the outcome domain of interest, (2) the validity and reliability of the outcome measure, (3) the interpretation of the measurement score for clinical practice, and (4) the feasibility of implementing the measurement in practice. The results were interpreted in a similar manner to the thresholds for the outcome domains. At the fourth meeting, the working group discussed how to enhance the outcome measures. Feedback regarding the outcome measures was further analysed and discussed by the research team at an additional session (see Fig. 1, research team meeting 2). At the fourth voting round the members of the working group were asked to indicate their agreement with the proposed changes to the outcome measures.

#### Phase 4. Selection of case mix factors

A targeted literature search was conducted by the research team to provide a long list of case mix factors extracted from literature and guidelines (see Fig. 1, search 3 and supplementary material 1). The working group was then asked to add to this long list of case mix factors if they considered it necessary (see Fig. 1, survey 4). Consensus on a minimal set of case mix factors was reached after discussion by the research team (see Fig. 1, research team meeting 3). The selection of the minimal set of case mix factors was based on the influence of the factors on the selected outcomes. The final standard set, including the minimal set of case mix factors, was shared with the working group for final approval.

### Role of the researchers and ethical considerations

All authors are experienced researchers in the field of occupational health and/or human-centred design. All participants signed an informed consent form and received compensation in return for their participation. The Medical Ethics Committee of the Amsterdam University Medical Center declared that the study design did not require comprehensive ethical review, as the Medical Research Involving Human Subjects Act did not apply to this study (Reference number: W22_304 # 22.382).

## Results

### Working group composition and response rates

The working group comprised 17 members, of which 6 were patients and 11 healthcare professionals. The patients’ diagnoses included various types of CVD (*n* = 2 cardiac arrhythmia, *n* = 1 coronary artery spasms, *n* = 1 heart valve disease, *n* = 2 aortic disease). At the time of diagnosis one patient was self-employed, four were contracted employees and one was a temporary worker. At the moment of this study, two were fully working, two were partly working and two were not working. The group of healthcare professionals included two insurance physicians, one working for the Dutch Social Security Agency and one working in the private sector, an occupational physician specialising in cardiovascular issues, a labour expert employed by both the Dutch Social Security Agency and a reintegration agency, a clinical physiotherapist involved in cardiovascular rehabilitation, an occupational physiotherapist, a nurse specialising in cardiology, a general practitioner, a psychologist employed by the Dutch Social Security Agency, a reintegration coach and a cardiologist. See Table 1 for further characteristics of the working group. In total the average attendance rate during the working group meetings was 85.3%, and the response rate for all voting rounds was 100%.

**Table 1:** Characteristics of the working group (n = 17)

Variable	Mean (SD) or n (percentage)
**Working group (n** = **17)**	
Age	50.7 (9.9)
Gender (male)	8 (47.1%)
**Patients (n=6)**	
Age	51.5 (7.8)
Gender (male)	1 (16.7%)
Time since diagnosis (years)	3.2 (1.6)
Type of CVD	
* Cardiac arrhythmia*	2 (33.3%)
* Coronary artery spasms*	1 (16.7%)
* Heart valve disease*	1 (16.7%)
Aortic disease	2 (33.3%)
Employment status at moment of diagnosis	
* Working fulltime*	2 (33.3%)
* Working part time*	3 (50.0%)
* Not working*	1 (16.7%)
Type of work arrangements	
* Self-employed*	1 (16.7%)
* Contracted employee*	4 (66.6%)
* Temporary worker*	1 (16.7%)
Current employment status	
* Fully working*	2 (33.3%)
* Partly working*	2 (33.3%)
* Not working*	2 (33.3%)
Job sector	
* Education and training*	1 (16.7%)
* Engineering, production and construction*	1 (16.7%)
* Healthcare and wellbeing*	2 (33.3%)
* Security and public administration*	2 (33.3%)
Present comorbidities	
* Musculoskeletal*	1 (16.7%)
* Neurological*	3 (50.0%)
* None*	2 (33.3%)
**Professionals (n = 11)**	
Age	50.3 (11.2)
Gender (male)	7 (63.6%)
Years of work experience	13.4 (9.7)

### Phase 1. Establish the scope and target population

The working group reached the consensus that the proposed scope of the standard set should be aligned with the following objectives: firstly, to assist healthcare professionals in addressing the individual needs of patients related to work participation, and secondly, to enhance the engagement of these patients in their own work-focused care process. In addition, the working group reached consensus on the proposed target population, adhering to the definition of CVD as outlined by the World Health Organization [26].

### Phase 2. Selection of outcome domains

**Fig. 2:**
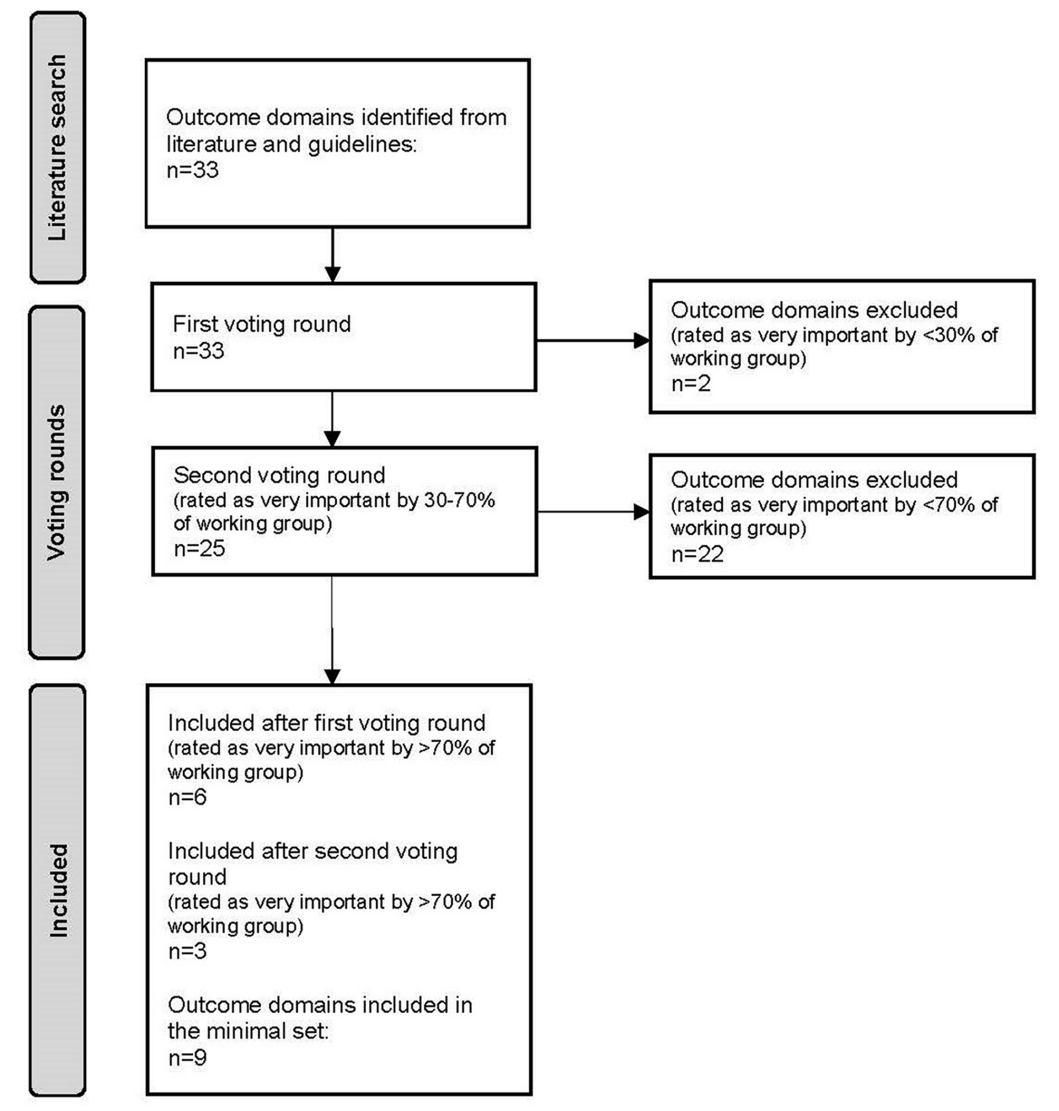
Flow diagram of the selection of the outcome domains. ‘Very important’ = 7–9 score on a 1–9 Likert Scale

Based on a literature search, 33 outcome domains were identified and subsequently subdivided into 5 categories based on the ICF model (see supplementary material 3) [25]. These were: (1) work factors (*n* = 5), (2) work ability (*n* = 4), (3) personal factors (*n* = 9), (4) external factors: work-focused healthcare (*n* = 12), and (5) external factors: social and work environment (*n* = 3). The working group reached consensus on 9 outcome domains rated as being most important in the first two voting rounds (see Fig. 2 and supplementary material 3). The final 9 outcome domains comprised: (1) work participation, (2) physical work ability, (3) mental work ability, (4) suitable work, (5) support from the working environment, (6) flexibility of the working environment, (7) communication towards the patient, (8) person-centredness, and (9) interdisciplinary communication. The definitions of these outcome domains can be found in Table 2. Key points of discussion are listed below.

**Table 2:** Proposed standard set of most important work-related outcomes to be used in practice for patients with cardiovascular disease

Part of questionnaire* and included outcome domains	Definitions of the outcome domains	Origin of chosen outcome measures*	Items (*n*)*
**Part 1 – Performance in paid work**			
Work participation	Extent to which the patient participates in work, such as having a job, number of hours and type of work.	All items of the ‘return to work’ domain from the standard set for patient with hand and wrist conditions [28] were included and adjusted to the context of CVD.	6
**Part 2 – Work ability**			
Physical work ability	The extent to which the patient can physically perform work.	The Work Ability Score (WAS) was specified for general, physical, mental and energetic work ability [29].	4
Mental work ability	The extent to which the patient can mentally perform work.		
**Part 3 – Suitable work**			
Suitable work	Having suitable work that matches the patient’s possibilities and limitations.	The fourth out of seven items of the Work Ability Index (WAI) [31] and the full Output Demand Scale of the Work Limitations Questionnaire (WLQ) [30] were both included.	6
**Part 4 – Work environment**			
Support from the work environment	The extent to which the work environment is involved and supportive for the individual.	A single item was derived from the 17-item first part on sociographic data and background information of the Work rehabilitation questionnaire (WORQ) [33]. The wording and response options were adjusted, including the addition of an additional question stating the context.	2
Flexibility of the work environment	The extent to which the work environment is able to take over tasks and offer adjustments in work.	A single item was derived from the 20-items ‘my supervisor’ scale of the Support for Workers with a Disability Scale (SWDS) [32]. The wording and response options were adjusted.	1
**Part 5 – Person-centredness**			
Communication towards the patient	The extent to which the patient experiences to be included in the flow of information within work-focused healthcare.	All items of the CollaboRATE questionnaire for patients 10-point scale [34] were included and adjusted to the context of work and health.	3
Person-centredness	Extent to which the patient feels that they are being treated correctly and that attention is paid to their personal situation.		
**Part 6 – Interdisciplinary communication**			
Interdisciplinary communication	The way in which information is exchanged between professionals involved in work-focused healthcare.	Self-developed item.	1

*The 23-item patient-reported questionnaire (Value@WORK-Q23) can be found in supplementary material 5

#### Work participation

Although during the first survey the outcome domain work disability was immediately appointed as very important by more than 70% of the participants, defining this outcome domain proved challenging. The insurance physicians involved, representing both the Dutch Social Security Agency and the private sector, highlighted that work disability, as defined in the realm of insurance medicine practice, entails a comprehensive assessment of earning capacity based on established functional capabilities. Patients in the working group expressed their perception that work disability, as defined within the practice of insurance medicine, carries a legal connotation with negative implications. They argued that this definition did not align with the scope of this standard set. Therefore, to contextualise work disability appropriately, the working group discussed what they considered most important within the scope of this standard set. They collectively agreed it was especially important to delineate the context of work participation. Therefore, the working group decided to reframe this outcome domain, by no longer referring to it as ‘work disability’ but as ‘work participation’.

#### Work ability

Four outcome domains related to work ability were included in the long list of outcome domains: physical work ability, mental work ability, sustainable recovery work ability, and social work ability. While the physical and mental work ability were promptly appointed as very important, the working group engaged in an extensive discussion regarding the importance of sustainable recovery work ability. However, consensus on this outcome domain was not reached.

#### Suitable work

Similar to the discussion regarding work participation, defining suitable work also proved to be challenging due to different interpretations among the members of the working group. Some members, including the occupational physician and the labour expert, interpreted suitable work in terms of what would be appropriate for a specific patient rather than focusing on the patient’s current work situation. Following a thorough discussion, the working group reached consensus that the focus should be on the existing work situation. They noted that the outcome becomes irrelevant for patients who do not have an active work environment.

#### Person-centredness

During the working group discussions, several patients emphasised that the term ‘patient-centredness’ felt overly restrictive in defining their experiences and carried a negative connotation. They expressed the need for a broader perspective that encompasses all aspects of being human. Consequently, one of the professionals proposed the term ‘person-centredness’, which was welcomed by the entire working group.

#### Personal factors

While the category personal factors included nine outcome domains, none of these were included in the standard set. The working group identified and discussed the matter. However, ultimately they concluded that none of the outcome domains in this category stood out as being more important than any other.

### Phase 3. Selection of outcome measures

A wide array of measurement instruments that could potentially measure the included outcome domains were identified by means of a literature search. When voting, the working group unanimously agreed on all proposals by the research team on how to measure each of the outcome domains (see supplementary material 4). Considering the suggestions for improvement by the working group, consensus was reached on a 23-item questionnaire for measuring the nine outcome domains. The measurement instrument for each of the outcome domains can be found in Table 2. The 23-item patient-reported questionnaire, called the Value@WORK-Q23, can be found in supplementary material 5. Significant discussion points, considerations and final decisions for selecting the outcome measures of all nine outcome domains are listed below.

#### Work participation

In the search for a suitable instrument to measure *work participation*, the research team found a core set that considered current employment status, work participation, and time to return to work to be the most important aspects when measuring work participation [27]. An earlier standard set for patients with hand and wrist conditions had included an outcome on return to work including outcome measures regarding these three aspects [28]. This original questionnaire on the hand and wrist was slightly adjusted to align with our focus on CVD.

#### Work ability

The Work Ability Score (WAS) was identified as an instrument to assess *generic work ability, physical work ability and mental work ability*, in accordance with the previous literature [29]. However, the patients in the working group indicated that energy levels and fatigue can significantly impact perceived work ability. Both patients and healthcare professionals concurred that energetic work ability cannot be adequately captured by measures regarding physical or mental work ability alone, as it is an independent aspect of work ability. Therefore, it was decided to incorporate a distinct question regarding energetic work ability within the domain.

#### Suitable work

In the search for a suitable measurement instrument for the outcome domain *suitable work*, the research team found multiple measurement instruments, each evaluating different aspects of suitable work. Consequently, to acquire a comprehensive understanding of the outcome suitable work, questions from two measurement instruments were combined: the fourth question from the Work Ability Index (WAI) and the Output Demand Scale from the Work Limitations Questionnaire (WLQ) [30, 31].

#### Support from & flexibility of the working environment

The working group discussed whether the two outcome domains *support from the working environment* and *flexibility of the working environment* should be considered as separate definitions, each requiring different measures. The patients expressed the opinion that these indeed differ, and therefore required different measures. In the view of the patients, support from the working environment is the social part of the support, including the involvement of the working environment., while flexibility is more the practical side, including the extent to which work tasks can actually be adjusted. Nevertheless, to maintain a logical structure within the standard set, the working group recommended combining the two outcome domains into one theme, but including both measures. For support from the working environment one question was selected from the Work Rehabilitation Questionnaire (WORQ) and for flexibility one question from the Support for Workers with a Disability Scale (SWDS) was selected [32, 33]. To interpret the answer on these measures, a self-developed question was added to quantify the extent to which support from the working environment is needed. These two outcome domains are irrelevant for those patients without an active work environment.

#### Communication towards the patient & person-centredness

For the outcome domains *communication towards the patient* and *person centredness*, the research team found that both outcome domains can be properly measured by the collaboRATE questionnaire. The collaboRATE questionnaire is a patient-reported measure for shared decision-making, including three questions relating to the effort made by the healthcare professional to understand the health issue, listen to the things that matter most about the health issue, and include what matters most to the patient [34]. These three questions transcended our previously established definitions of the two outcome domains, which led to the decision to merge the two outcome domains. However, the original version of the collaboRATE lacks a work-related focus so with the permission of the developer, the collaboRATE was slightly adjusted to include the work-related focus for our purpose.

#### Interdisciplinary communication

In the search for a suitable measurement instrument for patients with regard to their experiences of the *communication between professionals*, no instruments were found. Therefore, the research team suggested adding a self-developed question, which was discussed and refined by the working group.

#### Order in patient-reported standard set

In addition to the outcome domain-specific discussions, the working group suggested a specific order to present the outcome measures in the patient-reported questionnaire. This proposed order was based on their understanding of the relation between the outcome domains and their measures. They suggested initiating the list with *work participation* and *work ability*, followed by *suitable work* and *support & flexibility from the working environment* as these outcomes are all closely related to the current work situation. Lastly, they recommended connecting the three outcome domains targeting *person centredness* and *communication*.

### Phase 4. Selecting case mix factors

The literature search identified a total of 21 case mix factors. These factors were subdivided into 3 categories including 7 demographic factors (e.g. age and gender), 7 disease specific factors (e.g. diagnosis and comorbidities), and 7 organisational work factors (e.g. type of employment contract and sector) (see supplementary material 6). Based on the input by the working group, the long list was supplemented with two additional case mix factors: the presence of depression (a disease specific factor) and previous periods of work disability (a work factor). Ultimately, the research team reached consensus on the importance of nine case mix factors. These case mix factors comprised four demographic factors, i.e. age, gender, educational level and postal code, two disease specific factors, i.e. type of CVD and comorbidities influencing work participation, and three work factors, i.e. work status prior to CVD, workload and previous periods of work disability. All members of the working group agreed on this selection. All definitions of the case mix factors are shown in Table 3. The items proposed for measuring these case mix factors can be found in supplementary material 7.

**Table 3:** Proposed minimal set of case mix factors to be able to compare the most important outcome domains on a group level

Category	Case mix factor	Definition
Demographic	Age	Age of the patient.
	Gender	Gender of the patient.
	Education	The highest educational level the patient has completed.
	Postal code	The letters and digits assigned to the geographical area the patient lives in. The postal code may be associated with a certain socio-economic status.
Disease specific	Type cardiovascular disease	The type of cardiovascular disease diagnosis a patient has received.
	Comorbidities influencing work participation	The presence of one or more additional conditions or diseases that have an influence on the work participation of the patient.
Work	Work-status prior to cardiovascular disease	If and for how many hours, the patient was working in a paid job at the time of the diagnosis of the cardiovascular disease.
	Workload	How much capacity the patient needs to perform current paid work.
	Previous periods of work disability	Any periods in the past during which the patient was unable to work due to a disability or health-related issue.

## Discussion

With an interdisciplinary group of (occupational) healthcare professionals and patients, we developed a standard set of key work-related outcomes for patients with CVD to be used in the practice of work-focused healthcare. Consensus on which are the nine most important outcome domains is reached: (1) work participation, (2) physical work ability, (3) mental work ability, (4) suitable work, (5) support from the work environment, (6) flexibility of the work environment, (7) communication with the patient, (8) person-centredness, and (9) interdisciplinary communication. For each of these outcome domains, consensus was reached on how to measure them, resulting in a 23-item patient-reported questionnaire. This questionnaire was called the Value@WORK-Q23. The Value@WORK-Q23 was complemented by nine case mix variables, consisting of demographic-, disease-, and work factors. It is important to acknowledge that this set does not encompass all outcomes that are significant to this patient population. Our goal was to develop a minimal set of key work-related outcomes in order to reduce the registration burden during data collection [14]. To our knowledge, this is the first standard set of patient-centred work-related outcome measures for patients with CVD, originating from the principles put forward by the value-based healthcare concept [12].

It is envisioned that this newly developed work-focused standard set will complement existing disease-specific standard sets. For instance, the disease-specific standard set for coronary artery disease does not yet integrate work-related outcomes [21]. By incorporating this work-focused standard set alongside disease-specific ones in daily healthcare practice, healthcare professionals will potentially gain better insight into the patient’s full personal situation, including their work situation. This additional insight helps healthcare professionals better meet the patient’s work-related needs [17], which is essential for improving the patient’s health-related quality of life [35]. Additionally, it has been found that completing patient-reported questionnaires encourages patients to reconsider their personal circumstances [36]. Our work-focused set may enhance the patient’s engagement in their work-focused healthcare process, and support work-related shared decision-making [37]. Engaging patients by addressing their responses to the questionnaire may, in turn, also enhance their health-related quality of life [38]. Given the heterogeneity in our target population, it should be acknowledged that not all outcome domains are equally relevant or applicable to all patients, as their individual work circumstances and work status vary widely.

The literature underscores the importance of the outcome domains; for instance, work participation was highlighted as a key outcome in previously developed patient-centred standard sets [28, 39]. Additionally, workplace accommodations and attitudes have been identified as influential factors affecting work participation, and have shown to influence the quality of care following stroke [40]. Furthermore, in a previous study patients emphasised the importance of person-centredness, effective information exchange, clear professional to patient communication, and interdisciplinary collaboration among healthcare professionals [11].

Somewhat surprisingly, no personal factors were included in our standard set. Consistent with the literature, our working group acknowledged the importance of the personal factors identified in relation to work participation [41]. However, the working group blamed the lack of consensus on the diverse and individually determined nature of personal factors, in which also the measurability and influenceability of these personal factors was questioned by healthcare professionals. Therefore, we suggest the personal factors should be candidate outcomes, and their importance should be considered on an individually patient basis.

### Methodological considerations

A significant strength of our standard set is that we adhered to the standardised and comprehensive approach used by ICHOM in developing over 40 person-centred standard sets [42]. However, the cut-off value for inclusion and exclusion of outcome domains and measures varied greatly between the different ICHOM studies [43]. Therefore, we chose our inclusion and exclusion rates of 70% and 30% respectively, based on averages found in the literature (66-80%; 0-50%) [24, 43]. The 30% exclusion rate resulted in two outcome domains being omitted after the first voting round (supplementary material 3). We believe that including these two outcome domains in the second voting round would not have impacted the final set. Similarly, an exclusion threshold of 50% would not have impacted the final set (supplementary material 3). Four outcome domains were included in the final set based on consensus scores between 70% and 80% (supplementary material 3). A stricter inclusion threshold of 80% could have resulted in fewer outcome domains being included in the final set. However, we support our decision to use a 70% inclusion threshold, as it ensured that the number of outcome domains included were comprehensive yet manageable [14].

Another strength of our study is the recognition of diversity of our patient population. In line with our commitment to incorporate a variety of patient perspectives, six of our working group members were patients (35%), surpassing the typical 25% representation in ICHOM working groups [42]. This relatively high percentage reflected our dedication to patient-centred research. Our patient representatives came from diverse backgrounds, including different types of work arrangements, employment statuses and types and stages of CVD, ensuring a broad representation of the CVD population. However, in the development of another standard set [23], an additional patient advisory group (*n* = 300+) was involved alongside the working group, where they rated the importance of each proposed outcome. This input was made transparent to the working group, enabling them to incorporate this information into subsequent discussions and voting. While our working group had a relatively high percentage of patients directly participating in the consensus process, the ratings from a larger patient group were not assessed. Although the inclusion of such an additional patient advisory group is not standard practice in the ICHOM method [42], we believe it could have helped our working group in considering the importance of the outcome domains. To ensure a holistic and inclusive perspective, we engaged stakeholders from all relevant professions throughout the patient’s work-focused healthcare process in the Netherlands [7]. Eleven healthcare professionals participated, providing balanced representation across various healthcare perspectives. This resulted in a working group of 17 members, which is a typical group size for developments of this kind (12–31) [43].

In addition, while most outcome measures in the developed standard set were selected from validated measurement instruments [30–32, 44, 45], several modifications were necessary to align the specific needs of work-focused healthcare. These adjustments included using single items, adapting measures to fit the context, or modifying response options. Such changes may have negatively impacted the external validity of the measures. The limited availability of appropriate measurement instruments for the included outcome domains once again underscores the novelty of measuring patient-reported work-related outcomes. Therefore, we had to rely on making adjustments to existing measures and to design new items to ensure the comprehensibility and manageability for patients, facilitating practical use. Therefore, the validity of the patient-reported questionnaire should be further investigated [46]. Future revisions of the standard set should consider newly validated outcome measures that require fewer adjustments to enhance overall validity.

### Implications for future research

While most standard sets are developed in an international setting, this particular standard set was specifically tailored for use in practice within the Netherlands. This decision was driven by notable differences in healthcare systems worldwide, particularly the distinct separation between the medical roles of clinical and occupational professionals in the Netherlands [47]. Consequently, it remains uncertain whether this standard set includes universally important outcomes and whether it can be effectively applied for patients in healthcare contexts outside the Netherlands. However, we suggest that some of the included outcome domains are likely transferable to healthcare settings outside the Netherlands. For instance, we believe that the outcome domains on work ability hold relevance for all patients experiencing work participation problems due to CVD, regardless the healthcare system. However, the importance of outcomes such as support and flexibility of the work environment, or interdisciplinary communication may be more strongly influenced by legislation and regulations and the professionals involved in the different healthcare systems. Nevertheless, international adoption of a standard set is desirable to facilitate cross-border learning and improvement. Therefore, future research is needed to determine the transferability of this standard set to other contexts and which adaptations are necessary. Collaborating with ICHOM partners could facilitate the development of an internationally applicable outcome set, in which our standard set serves as the foundation. The current standard set developed for use in practice aims to encompass the key work-related outcomes for individuals, particularly those living with CVD. However, work is a critical outcome for all adults managing health conditions within the working-age population [39]. It can be assumed that the results in the standard set are not only important for people with CVD, but that the set can be generically applied with minimal adjustments for people who experience work problems due to chronic illness. Therefore, future research should investigate whether key work-related outcomes vary across different medical conditions to determine the generalizability of our work-related standard set. As a next step, the added value of the newly developed standard set needs to be tested in practice, in order to assess its feasibility for implementation, use and impact. Hereby, it would also be interesting to explore the feasibility and impact of measuring all the included outcome domains across different healthcare settings. For example, in curative healthcare, where PROMs are already widely used, the standard set could be distributed alongside other existing PROMs. Adding the full standard set could impose a burden for both patients and professionals. Therefore, selecting a subset of domains from this standard se t that provide meaningful insights while minimizing the response burden for patients and administrative load for professionals may be essential for successful implementation. Additionally, studies should investigate the validity and reliability of these subsets to ensure they effectively capture essential patient-reported outcomes.

### Implications for practice

The work-related standard set developed in the present study serves to help healthcare professionals and policymakers to deliver value-driven care. The developed standard set aims for person-centred quality improvements by means of a dual strategy at both an individual and aggregate level. At an individual level, healthcare professionals gain insight into the patient’s answers, enabling discussion during consultations on those work-related topics that are most important for the patient. This empowers shared decision-making by considering the individual’s situation, their needs and preferences. It allows healthcare professionals to tailor care plans or return-to-work plans specifically for work-related concerns, thus enhancing patient engagement and satisfaction. At an aggregate level, healthcare institutions will be able to benchmark their performance against one another. Comparing institutions can reveal necessary quality improvements and facilitate learning across organisations. However, to enable such comparison between institutions, an infrastructure for sharing data, such as a registries, should be available. In the Netherlands, work-focused healthcare and curative care are two distinct medical domains [7], which poses challenges for using, implementing and deploying standardised outcome sets in an integrated, team-orientated manner. Therefore, until an infrastructure for sharing outcome data across healthcare domains is established, we recommend that healthcare institutions integrate the standard set into their own digital environments for use at the individual level and exchange aggregate data within departments or with partners already involved in existing digital care pathways.

## Conclusion

The newly-developed standard set measures key work-related outcomes for patients with CVD in practice. Using this work-related set in addition to existing disease-specific standard sets for CVD will facilitate high-value work-focused healthcare for this patient population.

## Electronic supplementary material

Below is the link to the electronic supplementary material.

Supplementary Material 1

## Data Availability

All data generated and analyzed during this study are included in this published article and its supplementary materials. Other information regarding the (methods of the) current study is available from the corresponding author on reasonable request.

## Abbreviations

CVD: Cardiovascular diseases
ICHOM: International Consortium for Health Outcomes Measurements
