# Supplementary material for: Development of a standard set of key work-related outcomes for use in practice for patients with cardiovascular disease: a modified Delphi study
Source: J Patient Rep Outcomes. 2024 Dec 18;8:147. doi: 10.1186/s41687-024-00825-6 (PMC11655757; doi:10.1186/s41687-024-00825-6)
Supplement: Supplementary file 1 — Supplementary Material 1 [file 41687_2024_825_MOESM1_ESM.docx]

Supplementary material

Development of a standard set of key work-related outcomes for use in practice for patients with cardiovascular disease: a modified Delphi study

Content

Supplementary material 1 – List of rapid search strategies used in the literature searches…..……..2

Supplementary material 2 – Long list of outcome domains…………...……………………….…....3

Supplementary material 3 – Results 2-round voting outcome domains……………………..…...….6

Supplementary material 4 – Results 2-round voting outcome measures...…………………..………7

Supplementary material 5 – The 23-item questionnaire measuring the nine key outcome domains..8

Supplementary material 6 – Long list of case-mix factors…..………………………………………12

Supplementary material 7 – The questions measuring the minimal set of case-mix factors…………13

Supplementary material 1 – List of rapid search strategies used in the literature searches

Search 1: Targeted literature search to identify work-related outcome domains

Search conducted through:

1. Rapid review of the results of previous studies conducted in the Value@WORK research line.
2. Rapid review of generic and CVD disease-specific ICHOM sets^a^
3. Rapid review of current generic and CVD disease-specific guidelines for professionals involved in work-focused healthcare^b, c^
4. Rapid literature review and search in PubMed [from inception to January 2023] on ‘outcome (domains)’ AND ‘work (participation / support)’

Search 2: Targeted literature search to identify measurement instruments

Search conducted through:

1. Rapid literature review and search in PubMed [from inception to April 2023] on ‘*Name outcome domain 1-9*’ AND ‘instrument’ OR ‘patient-reported’
2. Rapid review of existing PROMs (e.g. meetinstrumentenzorg.nl)
3. Rapid review of generic and CVD disease-specific guidelines for professionals involved in work-focused healthcare^b, c^

Search 3: Targeted literature search to identify case-mix factors

Search conducted through:

1. Rapid review of the results of previous studies conducted in the Value@WORK research line.
2. Rapid review of the case-mix factors included in the generic and CVD disease-specific ICHOM sets.
3. Rapid review of eneric and CVD disease-specific guidelines for professionals involved in work-focused healthcare^b, c^
4. Rapid literature review and search in PubMed [from inception to April 2023] on ‘*Name outcome domain 1-9’* AND ‘covariates’ OR ‘case-mix’

^a^ [Patient Centered Outcomes For Health Measures - ICHOM](https://www.ichom.org/patient-centered-outcome-measures/)

^b^ [NVAB Richtlijnen | NVAB](https://nvab-online.nl/richtlijnen/richtlijnen-NVAB)

^c^ [Richtlijnen | NVVG](https://www.nvvg.nl/richtlijnen/)

*The references used to compile the long lists can be found in appendices showing the long-lists below.*

Supplementary material 2 – Long list of outcome domains

| **Outcome domain (N=33)** | **Definition** |
| --- | --- |
| ***Work factors (N=5)*** | |
| **Current work participation** | Extent to which you participate in work, such as having a job, the number of hours and type of work. |
| **Financial status** | Your financial status, such as loss of income and presence of social disability benefit. |
| **Suitable work** | Having suitable work that matches your possibilities and limitations. |
| **Work disability** | When you are unable to participate fully or partially in a form of work for a certain (often longer) period of time. |
| **Sick leave** | Loss of work due to your illness, such as the duration or frequency of absence or the number of hours worked less. |
| ***Work ability (N=4)*** |  |
| **Physical work ability** | The extent to which you can physically perform work, it can be influenced by the disease or medication. |
| **Mental work ability** | The extent to which you can mentally perform work, it can be influenced by the disease or medication. |
| **Sustainable recovery of work ability** | Degree to which you manage to rebuild an maintain working hours. |
| **Social work ability** | Degree to which you manage to handle contacts in the workplace, such as expressing feelings, dealing with conflicts and being able to work together. |
| ***Personal factors (N=9)*** | |
| **Expectations regarding reintegration** | Expectations of the person with chronic illness in terms of reintegration possibilities, as expected future work. |
| **Motivation for performing work** | Motivation and intention of the person with chronic illness to perform work. |
| **Coping with regard to work** | To what extent the person with chronic illness can accept the new situation and the way in which the person with chronic illness approaches this and looks for ways to work in the new situation. |
| **Need for work-oriented support** | Extent to which the person with chronic illness needs work-focused support. |
| **Work-life balance** | Extent to which work is compatible with the personal life of the person with a chronic illness. |
| **Meaning through work** | Degree of experienced social interaction, usefulness, and integration in society through (the lack of) work. |
| **Experienced self-sufficiency in work** | The person with chronic illness’s sense of competence to perform work, as well as self-confidence in being able to perform work. |
| **Fear of job loss** | Fear of the person with a chronic illness about losing a job, having a relapse during reintegration and/or consequences of this on income. |
| **Participatory behaviour** | The activities the person with chronic illness undertakes to get to work and stay at work. |
| ***External factors: Work-focused healthcare (N=12)*** | |
| **Accessibility of work-focused**  **healthcare** | Extent to which the patient has the opportunity to use occupational care facilities. |
| **Coordination within work-focused**  **healthcare** | Extent to which coordination between different parties has been arranged. |
| **Flexibility of work-focused**  **healthcare** | Extent to which the patient experiences the possibility and autonomy to adapt the occupational care based to their own needs. |
| **Continuity with work-focused**  **healthcare** | Extent to which occupational care flows smoothly into each other. |
| **Interdisciplinary communication** | The way in which information is exchanged between professionals involved in work-focused healthcare. |
| **Communication towards the patient** | Way in which the patient is included in the information flows within work-focused healthcare and the patient’s experiences with this. |
| **Transparency in communication** | Degree to which the patient is aware of information flows between different professionals. |
| **Experienced pressure to return to**  **work** | Extent to which the patient experiences pressure by the environment to return to work. |
| **Understanding of the law and**  **regulations** | Degree to which the patient has knowledge of the laws and regulations within the work-focused healthcare process. |
| **Insight into the occupational care**  **process** | Degree to which the patient knows what to expect, such as which steps to follow. |
| **Usability of support and advice** | Extent to which the patient experiences the occupational care received as useful and finds it useful in terms of content. |
| **Client focused** | Extent to with the patient feels that they are being treated correctly and that attention is paid to their personal situation. |
| ***External factors: Social and work environment (N=3)*** | |
| **Support from the social environment** | To what extent the social environment (family and friends) is involved and supportive of the person with chronic illness with regard to work and reintegration. |
| **Support from the work environment** | To what extent the work environment is involved and supportive for the person with chronic illness. |
| **Flexibility within the work**  **environment** | To what extent the work environment is able to take over tasks and offer adjustments in work. |

Sources of the outcome domains:

BG richtlijn chronische zieken en werk - Richtlijn_ChronischZiekenenWerk.pdf (nvab-online.nl)

BG richtlijn ischemische hartziekten - RL Ischemische Hartziekten 2020.pdf (nvab-online.nl)

Existing ICHOM Sets: [Patient Centered Outcomes For Health Measures - ICHOM](https://www.ichom.org/patient-centered-outcome-measures/)

Hagendijk, M. E., et al. (2023). Work-focused healthcare from the perspective of employees living with cardiovascular disease: a patient experience journey mapping study. *BMC public health*, *23*(1), 1765.

Hagendijk, M. E., et al. (2024). Patients’ Needs Regarding Work-Focused Healthcare: A Qualitative Evidence Synthesis. *Journal of Occupational Rehabilitation*, 1-19.

NVVG richtlijn participatiegedrag - [Boekje Participatiegedrag.indd (nvvg.nl)](https://www.nvvg.nl/files/1175/01_Boekje_Participatiegedrag.pdf)

NVVG richtlijn Chronische hartfalen - [Boekje COPD en CHF.indd (nvvg.nl)](https://www.nvvg.nl/files/1179/05_COPD_-_Chronisch_Hartfalen.pdf)

Ravinskaya, M., et al. (2023). Which outcomes should always be measured in intervention studies for improving work participation for people with a health problem? An international multistakeholder Delphi study to develop a core outcome set for Work participation (COS for Work). *BMJ open*, *13*(2), e069174.

Supplementary material 3 – Results 2-round voting outcome domains

| **Outcome domain (N=33)** | **Round 1**  **% very important (7-9)** | **Round 2**  **% very important (7-9)** | **Inclusion** |
| --- | --- | --- | --- |
| ***Work factors (N=5)*** | | |  |
| Current work participation | 64,7 | 64,7 | No |
| Financial status | 64,7 | 58,8 | No |
| Suitable work | **82,4** |  | **Yes** |
| Work disability | **70,6** |  | **Yes** |
| Sick leave | 64,7 | 35,6 | No |
| ***Work ability (N=4)*** | | |  |
| Physical work ability | **82,4** |  | **Yes** |
| Mental work ability | **76,5** |  | **Yes** |
| Sustainable recovery of work ability | 64,7 | 47,1 | No |
| Social work ability | 29,4 |  | No |
| ***Personal factors (N=9)*** | | | |
| Expectations regarding reintegration | 47,2 | 52,9 | No |
| Motivation for performing work | 52,9 | 47,1 | No |
| Coping with regard to work | 58,8 | 58,8 | No |
| Need for work-oriented support | 52,9 | 35,3 | No |
| Work-life balance | 41,2 | 47,1 | No |
| Meaning through work | 29,4 |  | No |
| Experienced self-sufficiency in work | 35,3 | 35,3 | No |
| Fear of job loss | 47,1 | 35,3 | No |
| Participatory behaviour | 52,9 | 64,6 | No |
| ***External factors: Work-focused healthcare (N=12)*** | | | |
| Accessibility of work-focused healthcare | 41,2 | 41,2 | No |
| Coordination within work-focused healthcare | 58,8 | 64,7 | No |
| Flexibility of work-focused healthcare | 58,8 | 52,9 | No |
| Continuity with work-focused healthcare | 35,3 | 23,5 | No |
| Communication between the professionals | 64,7 | **88,2** | **Yes** |
| Communication towards the patient | **70,6** |  | **Yes** |
| Transparency in communication | 47,1 | 23,5 | No |
| Experienced pressure to return to work | 47,1 | 41,2 | No |
| Understanding of the law and regulations | 17,6 | 35,3 | No |
| Insight into the occupational care process | 58,8 | 52,9 | No |
| Usability of support and advice | 41,2 | 17,6 | No |
| Patient-centeredness | **70,6** |  | **Yes** |
| ***External factors: Social and work environment (N=3)*** | | | |
| Support from the social environment | 35,3 | 29,4 | No |
| Support from the work environment | 58,8 | **88,2** | **Yes** |
| Flexibility within the work environment | 52,9 | **82,4** | **Yes** |

Supplementary material 4 – Results 2-round voting outcome measures

| **Outcome domain** | **Outcome measure*** | **Round 1**  % agree with the measure being the best option to measure the domain (7-9) | **Inclusion** | **Round 2**  % agree with the modifications (Yes) |
| --- | --- | --- | --- | --- |
| Work participation | *The work participation questionnaire developed by the ICHOM set for patient with hand and wrist conditions adjusted to the context of CVD* | **82,4** | **Yes** | **100,0** |
| Physical & mental work ability | *The work ability score (WAS) for general, physical, mental and energetic work ability* | **76,5** | **Yes** | **94,1** |
| Suitable work | *Fourth question of the Work ability index (WAI) + de output demand scale of the Work Limitations questionnaire (WLQ)* | **82,4** | **Yes** | **88,2** |
| Communication towards the patient &  person-centeredness | *CollaboRATE questionnaire adjusted to the context of work and health* | **88,2** | **Yes** | **82,4** |
| Support from the work environment | *Single question from the Work rehabilitation questionnaire (WORQ)* | **76,5** | **Yes** | **94,1** |
| Flexibility within the work environment | *Single question from the Support for Workers with a Disability Scale (SWDS)* | **82,4** | **Yes** | **94,1** |
| Communication between professionals | *Self-made question* | **70,6** | **Yes** | **100,0** |

*For references to the measurement instruments, see manuscript.

Supplementary material 5 – The 23-item questionnaire measuring the nine key outcome domains

***Please note:*** *This is an unofficial English translation of the Dutch Questionnaire with the aim to give our international readers insight into the type of questions included in the questionnaire. However, consensus with the working group was reached only on the Dutch version of the questionnaire. For the official Dutch version of the questionnaire, please contact the authors.*

**Part 1 (outcome domain 1 – work participation): Performance in paid work**

1. **Do you currently have a contract for paid work?**

Yes

No If no, continue to question 6.

1. **For how many hours per week do you currently have a contract for paid work?**

*If you are employed, enter the number of hours per week stated in your employment contract. If you work independently, complete a weekly estimate based on your experience.*

____________ hours per week

1. **Are you currently performing your job?**

*Due to your cardiovascular disease, you may be unable to work for a period of time. Are you currently performing your job? This can be in its entirety, or in the form of adapted tasks or, for example, fewer hours. Choose the answer that fits best:*

No, I'm not working at the moment. That is not due to my cardiovascular disease.

No, I'm not working at the moment. That is due to my cardiovascular disease.

On this date I called in sick: ______/______ *(MM/YYYY)*

Is there a date scheduled to return to work?  Yes  No

If the answer is yes:

This is the scheduled date to perform my job again: ______/______ *(MM/YYYY)*

Yes, I was reported sick but am now (partly) back to work.

On this date I called in sick: ______/______ *(MM/YYYY)*

On this date I started working again: ______/______ *(MM/YYYY)*

Yes, I have not previously been called in sick for my cardiovascular disease.

1. **If the answer to question 3 is ‘Yes’: How many hours per week do you currently work?**

____________ hours per week  Not applicable

1. **If the answer to question 3 is ‘Yes’: Can you perform all work tasks you were used to?**

Yes, I perform all my work tasks.

No, I do not (yet) perform the work tasks I was used to due to my cardiovascular disease.

No, I am not performing the work tasks I was used to, but for a different reason.

Not applicable

1. **How confident are you that you can return to or remain at work?**

A lot of confidence

Average confidence

Moderate confidence

No confidence

**Part 2 (outcome domain 2 & 3 – physical and mental work ability): Work ability**

*0 = unable to work at all*

*10 = the best work ability you have ever experienced*

1. **Do you feel that you are able to perform work?**
2. 1 2 3 4 5 6 7 8 9 10
3. **Do you feel that you are physically able to perform work?**

*Consider the physical capabilities of working, such as lifting, standing, bending and/or climbing (stairs).*

1. 1 2 3 4 5 6 7 8 9 10
2. **Do you feel that you are mentally able to perform work?**

*Consider your concentration, performing complex tasks or work under (time) pressure?* 1 2 3 4 5 6 7 8 9 10

1. **Do you feel you have enough energy to perform work?**

*Think about your endurance, or how quickly you get tired.*

0 1 2 3 4 5 6 7 8 9 10

*If you indicated in question 1 that you currently have no paid work, you may directly continue with part 5.*

**Part 3 (outcome domain 4 – suitable work): Suitable work**

**11. Does your cardiovascular disease make it difficult to perform your job?**

Please tick all the answers that apply to your situation:

☐ No, my condition does not make it difficult for me to perform my job.

☐ I can do my job, but I do notice that I have some complaints.

☐ I sometimes have to work slower or in a different way.

☐ I often have to work slower or in a different way.

☐ I can only work part-time.

☐ In my opinion, I can't work at all right now.

*If you previously indicated in question 4 that you are not currently performing your job, you may skip questions 12 to 16. You may continue to part 4.*

Please circle how difficult the following five requirements are for you in your work right now:

**12. Work fast enough**

Always difficult – usually difficult – sometimes difficult – rarely difficult – never difficult

Not applicable

**13. Finish work on time**

Always difficult – usually difficult – sometimes difficult – rarely difficult – never difficult

Not applicable

**14. Work without mistakes**

Always difficult – usually difficult – sometimes difficult – rarely difficult – never difficult

Not applicable

**15. Done what you are capable of**

Always difficult – usually difficult – sometimes difficult – rarely difficult – never difficult

Not applicable

**16. Handle workload**

Always difficult – usually difficult – sometimes difficult – rarely difficult – never difficult

Not applicable

**Part 4 (outcome 5 & 6: support from, and flexibility within the work environment): Work environment**

**17. How much support do you need from the people at your work?**

None A lot

0 1 2 3 4 5 6 7 8 9 10

**18. How much support you get from the people at your work?**

None A lot

0 1 2 3 4 5 6 7 8 9 10

**19. How well do the people at your work help you adjust your tasks, your hours, or your workplace? Is there room to adapt your work yourself?**

None A lot

0 1 2 3 4 5 6 7 8 9 10

**Part 5 (outcome domain 7 & 8: Communication towards the patient & person-centeredness): Person centeredness**

**20. How much effort is made to help you understand how your health affects your work situation?**

No effort was made Every effort was made

0 1 2 3 4 5 6 7 8 9 10

**21. How much effort is made to listen to the things that matter most to you about your health and work situation?**

No effort was made Every effort was made

0 1 2 3 4 5 6 7 8 9 10

**22. How much effort is made to include what matters most to you in choosing what to do next regarding your health and work situation?**

No effort was made Every effort was made

0 1 2 3 4 5 6 7 8 9 10

**Part 6 (outcome domain 9 – interdisciplinary communication): Interdisciplinary communication**

**23. Do you think that your healthcare providers cooperate well when it comes to your work situation?** No, not at all Yes, completely

0 1 2 3 4 5 6 7 8 9 10

Supplementary material 6 – Long list of case-mix factors

| Case-mix factor | Definition |
| --- | --- |
| *Demographic factors (N = 7)* |  |
| Age | The number of life years of the patient. |
| Gender | Gender characteristics of the patient. |
| Education level | The highest education that the patient has completed. |
| Money maker | Whether the patient is the breadwinner of the household. The breadwinner is the person in the household with the most important socio-economic position. |
| Household composition | Relationships of the patients with the people within their household. |
| Marital status | Whether the patient is married. |
| Income | Income of the patient, including salary or benefits |
| *Disease specific factors (N=7)* |  |
| Type of cardiovascular disease | The type of cardiovascular disease diagnosis a patient has. |
| Time since the cardiovascular diagnosis | How long the patient has been living with the diagnosis of the cardiovascular disease. |
| Type of medical treatment | The medical treatment the patient received/receives to treat the cardiovascular disease. |
| Medication use | Use of medication for the cardiovascular disease. |
| Existing comorbidities | Comorbidities of the patient in addition to the cardiovascular disease. |
| Pumping function | Pumping function of the heart. |
| *Organisatorische werkfactoren (N=7)* |  |
| Work status prior to diagnosis | Whether, and for how many hours, the patients was working in a paid job at the time of the diagnosis of the cardiovascular disease. |
| Type of contract | Type of contract that the patient had/has fort he paid job: permanent contract, temporary contract, temporary worker of self-employed. |
| Time since work participation problems | How long the patient has work participation problems due to their cardiovascular disease. |
| Type of work | The type of profession that the patient did/does in paid employment. |
| Heaviness of the work | How much capacity the patient needs to perform his/her paid work. |
| Size of the company | The number of employees of the company where the patient works. |
| Irregular shifts | Whether the patient’s work includes irregular shifts. |
| Job sector | Sector in which the patient is/was employed. |

Sources of the case-mix factors:

BG richtlijn chronische zieken en werk - Richtlijn_ChronischZiekenenWerk.pdf (nvab-online.nl)

BG richtlijn ischemische hartziekten - RL Ischemische Hartziekten 2020.pdf (nvab-online.nl)

Daniels, K., et al. "Development of an international, multidisciplinary, patient-centered Standard Outcome Set for Multiple

Gragnano, A., Negrini, A., Miglioretti, M., & Corbière, M. (2018). Common psychosocial factors predicting return to work after common mental disorders, cardiovascular diseases, and cancers: a review of reviews supporting a cross-disease approach. Journal of occupational rehabilitation, 28, 215-231.

Hagendijk, M. E., et al. (2024). Towards person-centred work-focused healthcare for people with cardiovascular disease: a qualitative exploration of patients’ experiences and needs. *Disability and rehabilitation*, 1-13.

McNamara, R. L., et al. (2015). Standardized outcome measurement for patients with coronary artery disease: consensus from the International Consortium for Health Outcomes Measurement (ICHOM). Journal of the American Heart Association, 4(5), e001767.

Mittag, O., Kolenda, K. D., Nordmann, K. J., Bernien, J., & Maurischat, C. (2001). Return to work after myocardial infarction/coronary artery bypass grafting: patients’ and physicians’ initial viewpoints and outcome 12 months later. Social Science & Medicine, 52(9), 1441-1450.

Ravinskaya, M., (2022). Extensive variability of work participation outcomes measured in randomized controlled trials: a systematic review. *Journal of clinical epidemiology*, *142*, 60-99.

Sclerosis: The SOS MS project." Multiple Sclerosis and Related Disorders 69 (2023): 104461.

Slebus, F. G., et al. (2012). Return to work after an acute coronary syndrome: patients’ perspective. *Safety and health at work*, 3(2), 117-122.

Supplementary material 7 – The questions measuring the minimal set of case-mix factors

1. **What is your gender?**

Female

Male

1. **Wat is your age?**

____________________________________

1. **What is the highest level of education you have completed?**

None

Primary education

Secondary school (VMBO, HAVO, VWO)

Secondary vocational education

Higher professional education

University (bachelor, master, PhD)

1. **What are the four digits of your zip code?**

**____________________________**(XXXX)

1. **What cardiovascular disease do you have?**

__________________________________________________________

1. **When (month/year) were you diagnosed with this cardiovascular disease?**

____________________________(MM/YYYY)

1. **Were you in paid work when you were diagnosed with your cardiovascular disease?**

*By paid work we mean an employed job. Or, if you are self-employed, an agreed assignment with an employment agency or client.*

Yes, I had a contract for paid work and also performed this work.

Yes, I had a contract for paid work, but did not perform this work due to health problems related to my cardiovascular disease.

Yes, I had a contract for paid work, but did not perform this work due to other health problems.

No, I did not have a contract for paid work.

1. **In addition to your cardiovascular disease, do you have other health problems that affect your participation in work?**

Yes

No

1. **If you answered ‘yes’ to the previous question: Which one?**

__________________________________________________________

__________________________________________________________

1. **Have you been unable to work for a long time in the past due to health problems?**

Yes

No

1. **If you answered ‘Yes’ to the previous question: How often and when have you been unable to work for a longer period of time due to health problems?**

__________________________________________________________

__________________________________________________________

1. **How would you rate the physical and mental exertion of you work?**

Light: Little physical and/or mental effort required.

Moderate: Moderate physical and/or mental effort required.

Heavy: High physical and/or mental effort required.
